# Prognostic significance and predictors of the system inflammation score in ovarian clear cell carcinoma

**DOI:** 10.1371/journal.pone.0177520

**Published:** 2017-05-12

**Authors:** Hongwei Zhang, Jiaqi Lu, Yingying Lu, Jiayi Zhou, Zehua Wang, Haiou Liu, Congjian Xu

**Affiliations:** 1 Department of Gynaecology, Obstetrics and Gynecology Hospital, Fudan University, Shanghai, China; 2 Shanghai Key Laboratory of Female Reproductive Endocrine Related Diseases, Shanghai, China; Seconda Universita degli Studi di Napoli, ITALY

## Abstract

Chronic inflammation is a well-known epidemiologic factor of ovarian clear cell carcinomas (OCCC), but has an uncertain role in prognosis. We developed a systemic inflammation score (SIS) based on preoperative serum albumin and neutrophil-to-lymphocyte ratio (NLR) for predicting progression-free survival (PFS) and overall survival (OS) in OCCC patients. A retrospective review was performed in 155 patients with OCCC undergoing primary debulking and chemotherapy at a single institute between 1995 and 2010. Cox regression models were fitted to analyze the effect of prognostic factors on PFS and OS. Harrell’s concordance index was calculated to assess predictive accuracy. The SIS consisting of serum albumin and NLR was retained as an independent indicator adjusting for traditional clinicopathological features. A high SIS was significantly associated with aggressive tumor behavior, platinum resistance, and served as an independent predictor of reduced PFS (*P* = 0.006) and OS (*P* = 0.019). The SIS had a good discrimination ability for the predictive PFS (c-index = 0.712) and OS (c-index = 0.722). We have developed a system inflammation score for predicting prognosis of OCCC patients, which may help stratify patients for postsurgical management.

## Introduction

Epithelial ovarian cancer remains the fourth leading cause of gynaecological cancer-associated death worldwide. Clear cell cancer accounts for 5% of ovarian cancers and is different to the other types of ovarian cancers in terms of biological and clinicopathological characteristics [1,2]. The prognosis for patients with stage I ovarian clear cell carcinoma (OCCC) is relatively good, while advanced stage of OCCC has a worse prognosis than serous ovarian cancers due to be resistant to the standard chemotherapy [3]. Accordingly, identification of clinical useful prognostic factors, in addition to common clinicopathological risk factors, to predict chemotherapy sensitivity and strengthen disease surveillance for OCCC could improve survival rates.

Ovarian clear cell carcinoma is partly derived from endometriosis, a chronic inflammatory disease [4,5]. Immune system contributes to chronic inflammation, malignant transformation, and the clinical course of the ovarian cancer [6]. The neutrophil-to-lymphocyte ratio (NLR) or platelet-to-lymphocyte ratio (PLR), a marker of systemic inflammation response, had been previously shown to serve as an independent prognostic marker for adverse clinical outcomes in ovarian cancer [7]. Moreover, a hypoalbuminemia was found to be associated with shortened overall survival in epithelial ovarian cancer [8]. However, there were few reports regarding the potential prognostic significance of these markers in ovarian clear cell carcinoma.

The aim of the present study is to assess the prognostic values of systemic inflammation response markers in patients of OCCC. We developed a systemic inflammation score (SIS) based on preoperative serum albumin and NLR, which was proven to be an independent predictor for PFS and OS. Moreover, integrating SIS and pathologic factors was improved predictive accuracy for patients of OCCC.

## Materials and methods

### Patients

This retrospective study reviewed the medical records of 155 patients with ovarian clear cell carcinoma who received operations at the Department of Gynecology in the Affiliated Obstetrics and Gynecology Hospital of Fudan University from 1995 to 2010. The study protocol was approved by the Ethics Committee of the Affiliated Obstetrics and Gynecology Hospital of Fudan University (No2015036), and the informed consent was obtained from all patients. The exclusion criteria included the presence of other malignancies, infection, trauma, coexisting hematological disease, autoimmune disorders or patients who failed to be followed up, or those without preoperative blood data available. All ovarian cancer patients followed up every 3 months for the first 2y and every 6 months for the next 3y. Gynecological examination, ultrasound, CT, chest-X ray and the serum concentration of CA-125 were evaluated at every visit. All the patients were staged according to guidelines of the International Federation of Gynecology and Obstetrics. Clinical and pathological data including age, surgical stage, distant metastasis, lymph node invasion, pre-treatment complete blood counts (Platelet, neutrophils and lymphocyte), serum albumin, and CA-125 were extracted from the retrospective medical records. Patients with ovarian endometriosis were pathological confirmed endometriosis histological contiguous to the tumor. NLR was defined as the absolute neutrophil count divided by the absolute lymphocyte count, and PLR was defined as the absolute platelet count divided by the absolute lymphocyte count. Moreover, we developed a novel systematic inflammation score (SIS) defined as follows: patients with decreased NLR and increased serum albumin were assigned score 0; patients with either increased NLR or decreased serum albumin were assigned score: 1; patients with increased NLR and decreased serum albumin were assigned score: 2. Overall survival (OS) was defined as the time (months) from the date of primary surgery to the date of individual death associated with the carcinoma/chemotherapy. Progression-free survival (PFS) was defined as the time (months) from the date of primary surgery to the date of disease recurrence or disease progression, and platinum resistance was defined as PFS less than 6 months. Patients were censored if they were lost to follow-up or if they show not progression or were still alive at the time of last follow-up. Follow up was updated in Feb 2015.

### Statistical analysis

Analysis was performed with MedCalc software (version 12.7.0.0; Mariakerke, Belgium) and Stata 12.0 (StataCorp, College Station, TX). Person X^2^-test or Fisher’s exact test was used to compare categorical variables and continuous variables were analyzed by Wilcoxon rank-sum test or Kruskal-Wallis test. The Kaplan-Meier method with log-rank test was used to compare survival curves. The Cox proportional hazards regression model was applied to perform univariate and multivariate analysis, and *P* > 0.10 was the removal criterion when performing backward stepwise variable deletions. The predictive accuracy of predictors was quantified by Harrell’s concordance index (*C*-index). All statistical tests were two sided and performed at a significance level of 0.05.

## Results

### Associations of PLR and NLR with clinicopathological characteristics

Geometric means (GM) and 95% confidence intervals (CI) for WBC, differential count, PLR and NLR by enrollment features and tumor characteristics were shown in Table 1. In general, neutrophil counts were significantly higher in patients with more aggressive tumor features including intraperitoneal metastasis, more residual tumor, present of ascites, high CA125 levels, and platinum resistance. Platelets counts were significantly correlated with FIGO stage, lymph node metastasis, present of ascites, high CA125 levels, decreased albumin levels, and platinum resistance. Elevated PLR and NLR were associated with platinum resistance.

**Table 1:** Patient characteristics in relation to WBC parameters, PLR and NLR.

Characteristic	N (%)	WBC (k/μl)	Neutrophils (k/μl)	Lymphocyte (k/μl)	Platelets (k/μl)	PLR	NLR
All cases	155 (100)	6.54 (6.22–6.86)	4.56 (4.28–4.84)	1.81 (1.58–2.05)	244.71 (231.32–258.10)	157.25(144.76–169.73)	3.02(2.64–3.40)
FIGO stage							
I	94 (60.6)	6.25 (5.84–6.65)	4.30(3.95–4.66)	1.86(1.56–2.16)	229.84(213.20–246.48)	145.28(129.44–161.11)	2.92(2.43–3.40)
II	16 (10.3)	6.59(5.61–7.57)	4.50(3.64–5.36)	1.80(1.07–2.52)	233.69(193.35–274.02)	157.10(118.72–195.48)	2.99(1.81–4.17)
III	40 (25.8)	7.05(6.43–7.67)	5.06(4.51–5.60)	1.71(1.25–2.16)	273.88(248.37–299.38)	182.52(158.25–206.80)	3.27(2.52–4.02)
IV	5 (3.2)	7.90(6.15–9.65)	5.54(3.99–7.08)	1.91(0.62–3.21)	326.20(254.05–398.35)	180.49(111.83–249.14)	3.06(0.95–5.18)
ANOVA *P*-value		0.073	0.080	0.954	**0.005**	0.079	0.894
Lymph node metastasis	136 (87.7)						
negative	19 (12.3)	6.42(6.09–6.76)	4.47(4.17–4.77)	1.81(1.55–2.07)	237.63(224.22–251.03)	154.58(141.29–167.88)	3.01(2.59–3.43)
positive		7.41(6.41–8.41)	5.17(4.34–6.00)	1.84(1.54–2.14)	295.42(244.94–345.91)	176.31(137.62–214.99)	3.07(2.39–3.75)
*t*-Test *P*-value		**0.043**	0.105	0.926	**0.005**	0.261	0.921
Intraperitoneal metastasis							
negative	121 (78.1)	6.29(5.97–6.63)	4.33(4.04–4.63)	1.84(1.56–2.13)	237.89(222.91–252.88)	149.56(136.24–162.89)	2.84(2.40–3.29)
positive	34 (21.9)	7.41(6.60–8.23)	5.36(4.67–6.05)	1.72(1.44–1.99)	268.97(239.25–298.69)	184.60(153.43–215.76)	3.64(3.01–4.27)
*t*-Test *P*-value		**0.004**	**0.003**	0.66	0.058	**0.021**	0.084
Residual tumor (cm)							
≤1	141(91.0)	6.42(6.12–6.73)	4.47(4.18–4.75)	1.82(1.57–2.07)	241.18(227.13–255.24)	153.71(140.85–166.57)	2.96(2.56–3.36)
>1	14 (9.0)	7.73(5.97–9.49)	5.48(4.18–6.78)	1.79(1.22–2.37)	280.21(235.14–325.28)	192.87(142.66–243.08)	3.61(2.59–4.62)
*t*-Test *P*-value		**0.019**	**0.04**	0.957	0.099	0.076	0.332
Ascites (ml)							
< 500	141 (91.0)	6.48(6.15–6.82)	4.47(4.18–4.76)	1.85(1.60–2.11)	239.55(225.67–253.42)	150.81(137.92–163.71)	2.90(2.50–3.30)
≥ 500	14 (9.0)	7.15(5.99–8.30)	5.47(4.45–6.49)	1.41(1.14–1.69)	296.71(250.66–342.77)	222.05(186.51–257.59)	4.18(3.25–5.12)
*t*-Test *P*-value		0.238	**0.044**	0.279	**0.015**	**0.001**	0.054
Post-menopausal							
No	58 (37.4)	7.05 (6.47–7.64)	4.99 (4.45–5.52)	1.77 (1.60–1.92)	260.79 (238.80–282.78)	166.94 (144.11–189.78)	3.26 (2.51–4.01)
Yes	97 (62.6)	6.24 (5.87–6.60)	4.30 (3.99–4.61)	1.84 (1.49–2.20)	235.09 (218.20–251.98)	151.45 (136.69–166.20)	2.87 (2.46–3.28)
*t*-Test *P*-value		**0.013**	**0.019**	0.746	0.066	0.237	0.329
CA125 level (U/ml)							
< 35	70 (45.2)	6.10(5.72–6.49)	4.10(3.77–4.43)	1.98(1.49–2.46)	219.31(203.33–235.30)	133.34(119.78–146.91)	2.62(2.12–3.13)
≥ 35	85 (54.8)	6.90(6.42–7.38)	4.93(4.51–5.35)	1.68(1.55–1.82)	265.62(245.87–285.38)	176.93(157.86–196.01)	3.34(2.80–3.89)
*t*-Test *P*-value		**0.013**	**0.003**	0.210	**<0.001**	**<0.001**	0.061
Endometriosis							
absent	106 (68.4)	6.49 (6.13–6.86)	4.56 (4.25–4.87)	1.82 (1.49–2.15)	252.31 (235.24–269.39)	163.95 (148.86–179.03)	3.0 (2.63–3.37)
present	49 (31.6)	6.65 (6.0–7.30)	4.55 (3.95–5.14)	1.80 (1.61–2.0)	228.27 (207.45–249.09)	142.75 (120.26–165.23)	3.05 (2.14–3.96)
*t-*Test P-value		0.654	0.960	0.942	0.099	0.119	0.907
Albumin (g/L)							
≤ 40	40 (25.8)	6.95 (6.22–7.68)	4.98 (4.37–5.59)	1.65 (1.45–1.85)	293.13 (260.38–325.87)	194.31 (166.13–222.49)	3.23 (2.84–3.62)
> 40	115 (74.2)	6.40 (6.05–6.75)	4.41 (4.10–4.72)	1.87 (1.57–2.18)	227.87 (214.90–240.83)	144.36 (131.22–157.49)	2.94 (2.45–3.44)
*t*-Test *P*-value		0.138	0.078	0.399	**<0.001**	**<0.001**	0.509
Platinum status							
sensitive	135 (87.1)	6.23 (5.97–6.50)	4.27 (4.03–4.50)	1.83 (1.57–2.08)	236.22 (222.42–250.02)	148.29 (136.87–159.72)	2.73 (2.44–3.02)
resistant	20 (12.9)	8.64 (7.14–10.13)	6.53 (5.24–7.82)	1.74 (1.31–2.18)	302.0 (261.72–342.28)	217.67 (162.30–273.04)	4.93 (2.81–7.06)
*t*-Test *P*-value		**<0.001**	**<0.001**	0.809	**0.001**	**<0.001**	**<0.001**

Abbreviations: FIGO = International federation of gynecology and obstetrics, CA125 = cancer antigen 125, PLR = platelet-lymphocyte count, NLR = neutrophil-lymphocyte count

### Associations of NLR, serum albumin and SIS with PFS and OS

We determined the association of clinicopathologic factors, albumin, PLR as well as NLR with PFS and OS by univariable analyses. We found that FIGO stage, lymph node metastasis, intraperitoneal metastasis, residual tumor, preoperative ascites, CA125, endometriosis, PLR, NLR, and albumin were significant prognostic indicators for PFS and OS, while age, post-menopausal had no significant association (Table 2). Based on multivariate analysis, the serum albumin (HR, 0.49; 95% CI, 0.27–0.91; *P* = 0.024), together with FIGO stage, residual tumor, preoperative ascites, and endometriosis were independent predictors for PFS. NLR (HR, 2.09; 95% CI, 1.06–4.13; *P* = 0.035) together with FIGO stage, residual tumor, and preoperative ascites were independent predictor for OS (Table 3).

**Table 2:** Univariate Cox regression analysis for PFS and OS of OCCC patients according to various clinicopathologic factors (n = 155).

Clinical variables	PFS HR (95%CI)	*P* value	c-index	OS HR (95%CI)	*P* value	c-index
Age (y)	1.00 (0.97–1.03)	0.977	0.519	1.00 (0.96–1.03)	0.898	0.521
FIGO stage		**< 0.001**	0.783		**< 0.001**	0.767
early stage (I-II)	Reference			Reference		
late stage (III-IV)	11.97 (6.74–21.25)			9.07 (4.99–16.50)		
Lymph node metastasis		**< 0.001**	0.625		**< 0.001**	0.601
negative	Reference			Reference		
positive	5.69 (3.13–10.32)			3.94 (2.04–7.63)		
Intraperitoneal metastasis		**< 0.001**	0.685		**< 0.001**	0.689
negative	Reference			Reference		
positive	6.15 (3.58–10.58)			5.83 (3.28–10.36)		
Residual tumor (cm)		**< 0.001**	0.597		**< 0.001**	0.6
≤ 1	Reference			Reference		
> 1	5.67 (2.97–10.80)			5.53 (2.80–10.91)		
Preoperative ascites (ml)		**< 0.001**	0.605		**< 0.001**	0.619
< 500	Reference			Reference		
≥ 500	6.25 (3.25–11.99)			6.43 (3.32–12.44)		
Post-menopausal		0.879	0.521		0.965	0.486
no	Reference			Reference		
yes	0.96 (0.56–1.64)			1.01 (0.57–1.81)		
Preoperative CA125 (U/ml)		**0.008**	0.614		**0.013**	0.614
< 35	Reference			Reference		
≥ 35	2.12 (1.22–3.68)			2.11 (1.17–3.80)		
Endometriosis		**< 0.001**	0.623		**0.002**	0.606
absent	Reference			Reference		
present	0.21 (0.09–0.50)			0.27 (0.11–0.62)		
PLR		**0.028**	0.587		**0.012**	0.601
≤ 141	Reference			Reference		
> 141	1.82 (1.07–3.09)			2.10 (1.18–3.73)		
NLR		**<0.001**	0.638		**< 0.001**	0.66
≤ 2.69	Reference			Reference		
> 2.69	2.71 (1.56–4.72)			3.18 (1.73–5.84)		
Albumin (g/L)		**<0.001**	0.648		**0.003**	0.638
≤ 40	Reference			Reference		
> 40	0.31 (0.18–0.53)			0.41 (0.23–0.73)		
Scoring system		**<0.001**	0.712		**<0.001**	0.722
0	Reference			Reference		
1	2.36 (1.22–4.58)	**0.011**		2.97 (1.44–6.11)	**0.003**	
2	6.73 (3.35–13.51)	**<0.001**		6.78 (3.13–14.68)	**< 0.001**	

Abbreviations: FIGO = International federation of gynecology and obstetrics, CA125 = cancer antigen 125, PLR = platelet-lymphocyte count, NLR = neutrophil-lymphocyte count, Scoring system = systemic inflammatory score, HR = hazard ratio, 95% CI = 95% confidence interval, c-index = Harrell’s concordance index

**Table 3:** Multivariate Cox regression analysis for PFS and OS of OCCC patients according to various clinicopathologic factors (n = 155).

Clinical variables	PFS			OS		
	HR (95%CI)	*P* value	c-index	HR (95%CI)	*P* value	c-index
**Model 1**			**0.868**			**0.827**
FIGO stage		**<0.001**			**<0.001**	
Early stage (I-II)	Reference			Reference		
Late stage (III-IV)	9.06 (4.82–17.05)			6.37(3.29–12.32)		
Residual tumor (cm)		**0.006**			**0.011**	
≤ 1	Reference			Reference		
> 1	2.76 (1.35–5.64)			2.58 (1.24–5.35)		
Preoperative ascites (ml)		**0.022**			**0.014**	
< 500	Reference			Reference		
≥ 500	2.53 (1.15–5.57)			2.55 (1.21–5.35)		
Albumin (g/L)		**0.024**				
≤ 40	Reference					
> 40	0.49 (0.27–0.91)					
NLR					**0.035**	
≤ 2.69				Reference		
> 2.69				2.09 (1.06–4.13)		
Endometriosis		**0.046**				
Absent	Reference					
Present	0.41 (0.17–0.98)					
**Model 2**			**0.874**			**0.838**
FIGO stage		**<0.001**			**<0.001**	
Early stage (I-II)	Reference			Reference		
Late stage (III-IV)	8.86 (4.71–16.69)			6.53 (3.33–12.83)		
Residual tumor (cm)		**0.018**			**0.014**	
≤ 1	Reference			Reference		
> 1	2.36 (1.17–4.79)			2.51 (1.21–5.21)		
Preoperative ascites (ml)		**0.033**			**0.012**	
< 500	Reference			Reference		
≥ 500	2.37 (1.08–5.22)			2.69 (1.24–5.81)		
Scoring system		**0.006**			**0.019**	
0	Reference			Reference		
1	2.05 (1.02–4.10)	**0.044**		2.79 (1.30–5.99)	**0.009**	
2	3.12 (1.36–7.15)	**0.008**		2.52 (0.99–6.42)	0.053	
Endometriosis		**0.04**				
Absent	Reference					
Present	0.40 (0.17–0.96)					

Abbreviations: FIGO = International federation of gynecology and obstetrics, CA125 = cancer antigen 125, PLR = platelet-lymphocyte count, NLR = neutrophil-lymphocyte count, Scoring system = systemic inflammatory score, HR = hazard ratio, 95% CI = 95% confidence interval, c-index = Harrell’s concordance index

**Fig 1:**
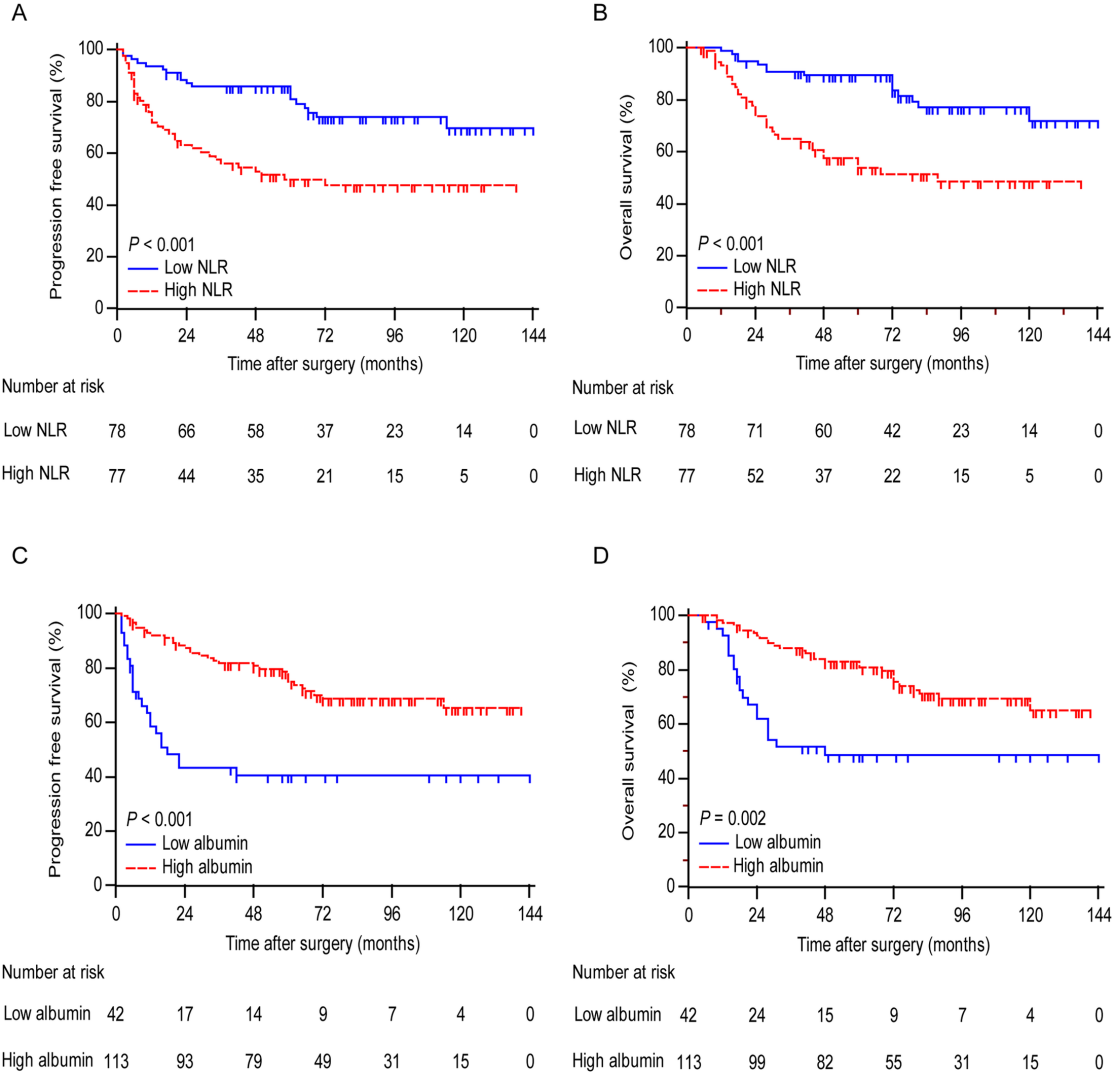
Analyses of progression-free survival and overall survival according to NLR and albumin in all patients. Curves show progression-free survival according to NLR (A) and albumin (C). Curves show overall survival according to NLR (B) and albumin (D). *P* values were calculated by log-rank test.

**Fig 2:**
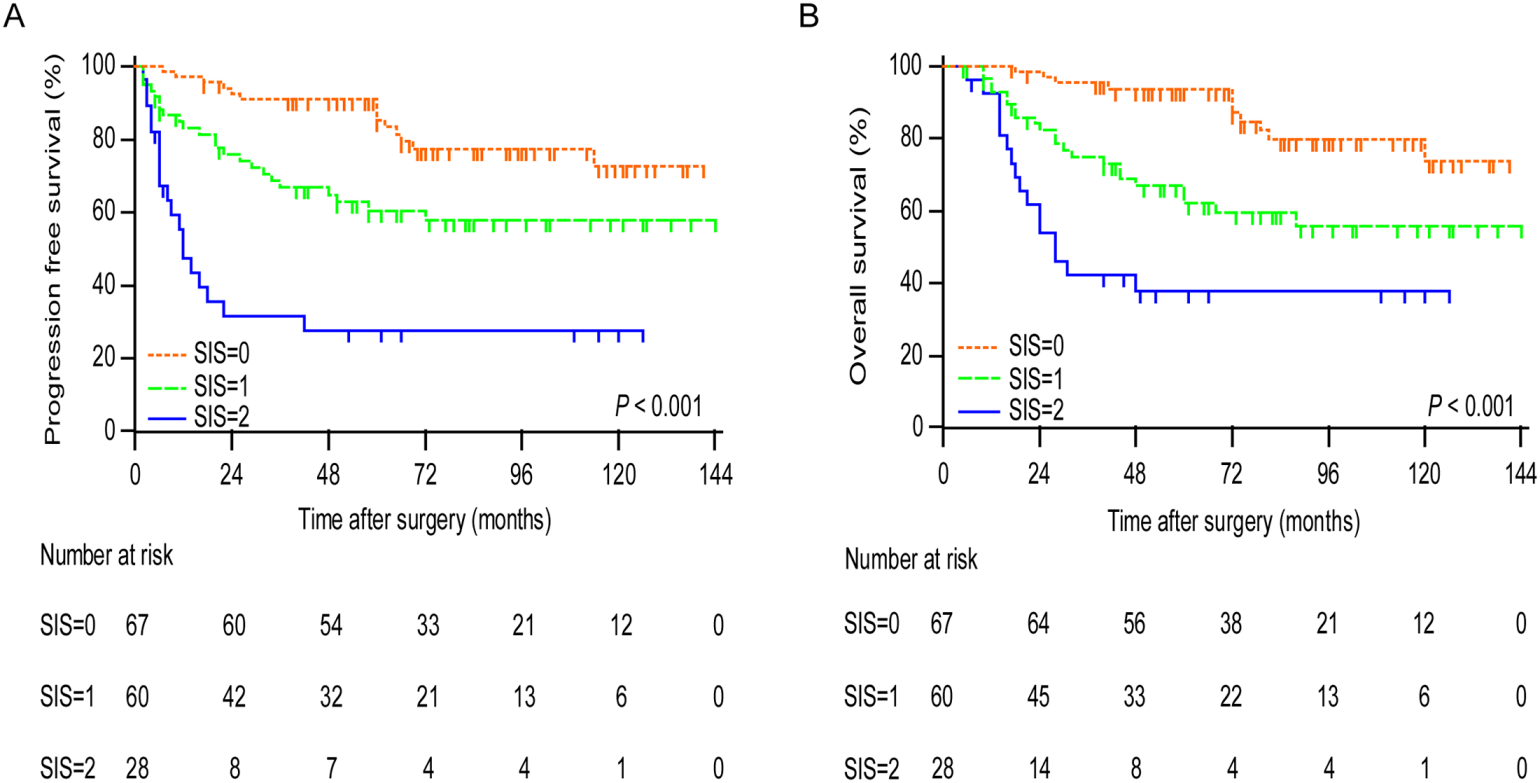
Analyses of progression-free survival and overall survival according to SIS in all patients. Curves show progression-free survival (A) and overall survival (B) according to SIS. *P* values were calculated by log-rank test.

Kaplan-Meier analysis indicated that the decreased serum albumin and high NLR were both associated with shorter PFS (*P* < 0.001 for both) and OS (*P* < 0.001 for NLR and *P* = 0.002 for albumin) (Fig 1). To further discriminate patients with different prognosis, we combined serum albumin and NLR levels to generate three subgroups. We established SIS defined as follows: patients with both elevated serum albumin and decreased NLR were assigned score 0; patients with either decreased serum albumin or increased NLR were assigned score 1 and patients with both decreased serum albumin and increased NLR were assigned score 2. Kaplan-Meier curves showed that high SIS was associated with shorter PFS and OS (*P* < 0.001 for both) (Fig 2).

In the univariate analysis, the SIS had prognostic significance for PFS and OS (*P* < 0.001 for both). The two most discriminatory univariate predictors of PFS were FIGO stage (c-statistic = 0.783) and SIS (c-statistic = 0.712). The two most discriminatory predictors of OS were FIGO stage (c-statistic = 0.767) and SIS(c-statistic = 0.722) (Table 2). The multivariate analysis demonstrated that SIS (*P* = 0.006 for PFS and *P* = 0.019 for OS) was the independent predictor for PFS and OS, together with FIGO stage, residual tumor and preoperative ascites, in addition to endometriosis for PFS. The *C*-index of model 1 for PFS was 0.868 and improved to 0.874 in model 2 by adding SIS, and the *C*-index of model 1 for OS was 0.827 and improved to 0.838 in model 2 by adding SIS (Table 3).

### Correlations between serum albumin, NLR, SIS and clinicopathological characteristics

The correlations between the serum albumin, NLR, SIS and clinicopathological characteristics were shown in Table 4. Decreased serum albumin and increased NLR were associated with advanced FIGO stage (*P* < 0.001 and *P* = 0.005, respectively), intraperitoneal metastasis (*P* < 0.001 and *P* = 0.01, respectively), more ascites (*P* = 0.013 and *P* = 0.002, respectively), elevated CA125 (*P* < 0.001 for both), and platinum resistant (*P* < 0.001 and *P* = 0.002, respectively). Additionally, decreased serum albumin was associated with absent of endometriosis (*P* = 0.015), and elevated NLR was associated with more residual tumor (*P* = 0.047). High SIS was more likely to be correlated with advanced FIGO stage (*P* < 0.001), lymph node metastasis (*P* = 0.01), intraperitoneal metastasis (*P* < 0.001), residual tumor (*P* = 0.005), more ascites (*P* < 0.001), increased CA125 (*P* < 0.001), absent of endometriosis (*P* = 0.006), and platinum resistance (*P* < 0.001).

**Table 4:** Clinicopathologic characteristics associated with albumin, NLR and SIS (*n* = 155).

Characteristics	Patients (n = 155)		Albumin		*P*	NLR		*P*	Scoring system			*P*
	Number	%	High (n = 115)	Low(n = 40)		High (n = 77)	Low (n = 78)		High (n = 28)	Medium (n = 60)	Low (n = 67)	
Age (y) †	155	100	50.82±8.08	52.35±8.98	0.317	50.27±9.01	52.14±7.53	0.163	51.52±9.27	50.98±8.82	51.26±7.55	0.958
FIGO stage					**<0.001**			**0.005**				**< 0.001**
I	94	60.6	80	14		38	56		7	35	52	
II	16	10.3	11	5		9	7		4	7	5	
III	40	25.8	23	17		27	13		14	16	10	
IV	5	3.2	1	4		3	2		3	2	0	
Lymph node metastasis					**0.044**			0.134				**0.01**
negative	136	87.7	105	31		64	72		21	52	63	
positive	19	12.3	10	9		13	6		7	8	4	
Intraperitoneal metastasis					**<0.001**			**0.01**				**<0.001**
negative	121	78.1	99	22		53	68		13	48	60	
positive	34	21.9	16	18		24	10		15	12	7	
Residual tumor (cm)					0.065			**0.047**				**0.005**
≤ 1	141	91.0	108	33		66	75		22	54	65	
> 1	14	9.0	7	7		11	3		6	6	2	
Preoperative ascites (ml)					**0.013**			**0.002**				**<0.001**
< 500	141	91.0	109	32		64	77		20	55	66	
≥ 500	14	9.0	6	8		13	1		8	5	1	
Post-menopausal					0.561			0.120				0.314
No	58	37.4	41	17		34	24		14	21	23	
Yes	97	62.6	74	23		43	54		14	39	44	
Preoperative CA125 (U/ml)					**<0.001**			**<0.001**				**<0.001**
< 35	70	45.2	62	8		24	46		5	23	42	
≥ 35	85	54.8	53	32		53	32		23	37	25	
Endometriosis					**0.015**			0.326				**0.006**
Absent	106	68.4	72	34		56	50		26	40	40	
Present	49	31.6	43	6		21	28		2	20	27	
Platinum status					**<0.001**			**0.002**				**<0.001**
Sensitive	135	87.1	108	27		60	75		17	51	67	
Resistant	20	12.9	7	13		17	3		11	9	0	

Abbreviations: FIGO = International federation of gynecology and obstetrics, CA125 = cancer antigen 125, NLR = neutrophil-lymphocyte count, Scoring system = systemic inflammatory score

^†^The results of continuous variables are expressed as mean±SD.

## Discussion

Although experimental evidence has extended multifaceted and sometimes paradoxical roles of neutrophils in cancer, the mounting clinical evidence assessing NLR makes sense that neutrophils promote, rather than inhibit cancer progression [9, 10]. NLR is indeed a reflection of the systemic inflammatory response that occurs in most cases of cancer patients. In the current study, a systemic inflammation scoring consisting of serum albumin and NLR have better discriminatory ability for predicting clinical outcomes compared with other clinicopathologic variables, except for FIGO stage.

The haematological markers such as the NLRs and PLRs can be used to predict clinical outcome and measure response to treatment, where high NLRs and PLRs have been associated with poor prognosis and failure to response to treatment [11]. However, the prognostic significance of NLR and PLR in ovarian cancer remains controversial [12–15]. Recently, PLRs have been used as an independent predictor of survival in patients with ovarian cancer [13]. In addition, the use of NLR could not only apply as a prognostic marker, but also aid in treatment choices in ovarian cancer [12,14,16,17]. Furthermore, variation in the reported NLR threshold used to assign patients to high-risk or low-risk cohorts complicates the application of a single NLR determination for patient’s prognostic prediction. The present study reported NLR cut-off point of 2.69 used as an independent predictor of OS in clear cell ovarian cancer patients.

Hypoalbuminaemia is the result of malnutrition and cachexia in cancer patients due to the host responses to the tumor and anti-tumor therapies. Therefore, hypoalbuminaemia also contributes to the increased mortality and provides prognostic significance [18]. Recently, hypoalbuminaemia has been used as an independent predictor of survival in ovarian cancer patients [8]. Furthermore, an integrated indicator based on hypoalbuminaemia and NLR was applied for the prediction of poor survival adjusted for other clinicopathologic characteristics in ovarian cancer [12]. Consistently with previous studies, we found out that hypoalbuminaemia alone acted as an independent predictor of PFS, while the systemic inflammation scoring consisting of hypoalbuminaemia and NLR used as an independent factor for predicting PFS and OS in clear cell ovarian cancer.

The relationship between high NLR or systemic inflammation score and disease outcome in clear cell ovarian cancer could probably be explained by the effects of neutrophils and lymphocytes on cancer progression. Neutrophils have been reported to promote tumorigenesis and progression by secreting tumor growth factors such as vascular endothelial growth factor (VEGF), hepatocyte growth factor [19], elastase [20], and MMP9 [21]. Moreover, neutrophils may exert an immunosuppressive function by suppressing the cytotoxic activity of immune cells such as lymphocyte and natural killer (NK) cells [22,23]. Furthermore, neutrophils mobilized by the primary tumors and suppressed NK cells-mediated clearance of tumor cells from initial sites of dissemination concurrently facilitating extravasation of tumor cells [24]. Neutrophils may initiate the metastatic potential of ovarian cancer cells as a result of cell-to-cell direct contact [25]. Tumor infiltrating lymphocytes (TIL) together with CD8+ T cells promote favorable prognosis in ovarian cancer [26], however, lymphocytopenia represent a state of decline in the cell-mediated immune which may limit tumor control following surgery and chemotherapy [27,28].

More recently, Kim et al. observed a NLR contributed to the predictive role in the PFS, and elevated NLR, PLR, and CA-125 were associated with advanced stage disease and platinum-resistance in patients with clear cell ovarian cancer [29]. On the one hand, the latter observation is in line with ours. On the other hand, we observed the presence of NLR correlated with OS instead of PFS. This discrepancy may be due to the different variables of prognostic factors. There are still limitations to this study. Firstly, it was a retrospective study with a small cohort. Secondly, there were some possible confounders that may influence systematic inflammatory response, such as smoking, the dietary regimen, and the use of oral contraceptive. Cigarette smoking has been observed as an interference with the immune system and trigger in immunosuppressive [30]. Dietary regimen may have anti-inflammatory effects, such as diets with fish-oil-derived fatty acids [31]. The use of oral contraceptive induces many changes in hematological markers and perturbs the macrophage function [32]. Thus, the systemic inflammatory scoring is a readily available and inexpensive biomarker, and its addition to established prognostic scores for clinical decision needs further large prospective investigation.
